# Disentangling Uric Acid and Renal Pathways in SGLT2 Inhibitor Effects After Acute Myocardial Infarction: A Retrospective Mediation Analysis

**DOI:** 10.3390/biomedicines14040842

**Published:** 2026-04-07

**Authors:** Ioana Maria Suciu, Călin Muntean, Laura Gaiță, Teodora Mateoc-Sîrb, Daliborca Cristina Vlad, Bogdan Timar, Dan Gaiță

**Affiliations:** 1 Doctoral School, “Victor Babeș” University of Medicine and Pharmacy, Eftimie Murgu Sq. No. 2, 300041 Timișoara, Romania; ioana-maria.suciu@umft.ro (I.M.S.);; 2 Institute of Cardiovascular Diseases Timisoara, Gheorghe Adam, 13A, 300310 Timișoara, Romania; 3 Research Center of the Institute of Cardiovascular Diseases Timișoara, Gheorghe Adam, 13A, 300310 Timișoara, Romania; 4 Department of Informatics and Medical Biostatistics, “Victor Babeș” University of Medicine and Pharmacy, Eftimie Murgu Sq. No. 2, 300041 Timișoara, Romania; 5 Department of Diabetes, Nutrition and Metabolic Diseases Clinic, “Pius Brînzeu” Emergency Clinical County University Hospital, 300723 Timișoara, Romania; 6 Department of Second Internal Medicine—Diabetes, Nutrition, Metabolic Diseases and Systemic Rheumatology, “Victor Babeș” University of Medicine and Pharmacy, Eftimie Murgu Sq. No. 2, 300041 Timișoara, Romania; 7 Department of Biochemistry and Pharmacology, Faculty of Medicine, “Victor Babeș” University of Medicine and Pharmacy, Eftimie Murgu Sq. No. 2, 300041 Timișoara, Romania; 8 Cardiology Department, “Victor Babeș” University of Medicine and Pharmacy, Eftimie Murgu Sq. No. 2, 300041 Timișoara, Romania

**Keywords:** SGLT2 inhibitors, acute myocardial infarction, uric acid reduction, renal function, cardioprotection

## Abstract

**Background/Objectives:** Sodium–glucose cotransporter-2 (SGLT2) inhibitors have demonstrated cardiovascular benefits beyond glycemic control, yet the specific biological pathways potentially linking SGLT2 inhibitor exposure to cardiovascular outcomes after acute myocardial infarction (AMI) remain incompletely characterized. Two biologically plausible pathways, serum uric acid (SUA) reduction and renal functional preservation, have been proposed, but not directly compared in a unified analytical framework. This study aimed to explore whether associations between SGLT2 inhibitor exposure and recurrent post-AMI outcomes may be more strongly linked to SUA reduction and to renal functional changes, using a hypothesis-generating causal mediation analysis. **Methods**: This retrospective observational cohort study included 142 consecutive patients hospitalized for AMI who underwent percutaneous coronary intervention (PCI) during the index hospitalization, reflecting standard-of-care management for AMI in this tertiary center. Patients were categorized by SGLT2 inhibitor exposure (*n* = 57) vs. controls (*n* = 85). Both diabetic (47.2%) and non-diabetic (52.8%) patients were included. The primary endpoint was change in SUA (ΔUA); the secondary endpoint was myocardial infarction (MI) recurrence. Causal mediation analysis with nonparametric bootstrap simulation tested both mechanistic pathways. **Results**: SGLT2 inhibitor therapy was associated with significant SUA reduction (ΔUA = −0.99 mg/dL vs. +0.56 mg/dL in controls; *p* < 0.001), consistent across diabetic and non-diabetic subgroups and independent of AMI recurrence. Each 1 mg/dL decrease in SUA was associated with lower odds of recurrent MI in the initial model (β = −0.25; *p* = 0.041). However, after incorporation of renal functional change, the uric acid-mediated pathway lost significance (ACME *p* = 0.462), whereas the renal-mediated pathway remained significant (ACME *p* = 0.038). Serum creatinine change emerged as the strongest independent predictor of MI recurrence (β = 2.22; *p* = 0.015). **Conclusions**: The findings are more consistent with a renal-mediated pathway than with an independent uric acid-mediated pathway in explaining the observed associations between SGLT2 inhibitor exposure and recurrent post-AMI outcomes. These hypothesis-generating results from a retrospective design warrant prospective validation.

## 1. Introduction

Recurrent cardiovascular events following acute myocardial infarction (AMI) remain a leading cause of morbidity, mortality, and healthcare burden worldwide. Despite improvements in revascularization and evidence-based pharmacological secondary prevention, the residual risk of recurrent myocardial infarction (MI) persists, particularly among patients with concomitant metabolic and renal risk factors [1,2].

Sodium–glucose cotransporter-2 (SGLT2) inhibitors, initially developed for glycemic control in type 2 diabetes mellitus (T2DM), have emerged as transformative agents in cardiovascular medicine. Landmark clinical trials, including EMPA-REG OUTCOME [3], DAPA-HF [4], and EMPEROR-Reduced [5], have consistently demonstrated reductions in cardiovascular mortality, heart failure (HF) hospitalization, and composite cardiovascular endpoints, with benefits extending to non-diabetic populations, and the European Society of Cardiology (ESC) now recommends SGLT2 inhibitors as first-line therapy for HF, irrespective of ejection fraction or diabetes status [6].

Furthermore, recent and ongoing trials in patients with AMI have started to highlight the potential benefits of SGLT2 inhibitors in the early post-AMI phase. The role of these agents has been increasingly investigated across a broad spectrum of study designs. Randomized controlled trials (RCTs), including DAPA-MI [7] and EMPACT-MI [8], have assessed their impact in post-AMI populations [9,10,11,12,13], while an expending body of observational studies [9,14,15,16,17,18,19,20,21,22,23,24,25,26,27,28,29] and several meta-analyses [30,31,32,33] have provided converging evidence suggesting beneficial effects on cardiovascular outcomes, ventricular remodeling, and overall prognosis after AMI.

While the clinical efficacy of SGLT2 inhibitors is well established, the precise biological mechanisms driving their cardiovascular benefits remain a subject of active investigation. Among the proposed mechanistic pathways, two have received substantial attention.

First, in the uric acid pathway, SGLT2 inhibitors enhance renal urate excretion through competitive inhibition of the URAT 1 and GLUT 9 transporters, leading to reductions in serum uric acid (SUA), a recognized cardiovascular risk factor [34,35,36]. While these urate-lowering effects are well established in diabetic populations, whether a comparable benefit persists in non-diabetic AMI patients remains uncertain. Therefore, we aim to evaluate the impact of SGLT2 inhibitor therapy on SUA homeostasis in patients with AMI, with particular focus on non-diabetic individuals. We hypothesized that SGLT2 inhibitors would reduce SUA through renal transport mechanisms even in the absence of overt hyperglycemia, supporting a pleiotropic benefit beyond glucose lowering. Emerging evidence supports the active role of SUA in cardiovascular pathology, and cardiovascular risk associated with SUA seems to appear evident even below the urate saturation threshold of 6.8 mg/dL, suggesting a crystal-independent pathogenic effect [37,38]. This evidence is supported by the URRAH (Uric Acid Right for Heart Health) project, which has highlighted a significant association between SUA and cardiovascular disease, supporting uric acid as both a clinically relevant biomarker and a potential mediator of cardiovascular risk [39]. Elevated SUA levels have been consistently linked to worse outcomes, including all-cause and cardiovascular mortality, as well as major adverse cardiovascular events (MACE) such as acute coronary syndrome (ACS), stroke, and peripheral artery disease [38,39]. Recent studies, including URRAH-related analyses, suggest that the traditional hyperuricemia thresholds may not be optimal for cardiovascular risk stratification [40,41,42,43,44,45]. Several lower SUA cut-offs have been proposed in relation to coronary and ischemic outcomes, as summarized in Table 1.

Second, the renal pathway, in which SGLT2 inhibitors restore tubuloglomerular feedback, reduce glomerular hyperfiltration and exert long-term nephroprotective effects that may independently lower cardiovascular risk [46,47]. Critically, these two pathways are physiologically interrelated, rather than mutually exclusive. Uric acid metabolism is intrinsically dependent on renal clearance, and both parameters are simultaneously influenced by SGLT2 inhibition. This collinearity complicates the interpretation of observational data and limits the ability of conventional regression analyses to attribute cardiovascular effects to one pathway vs. the other. To address this methodological challenge, causal mediation analysis offers a formal framework for decomposing total treatment effects into direct and indirect (mediated) components, enabling rigorous testing of specific mechanistic hypotheses [13,14].

The present study therefore aims to: (1) confirm the urate-lowering effect of SGLT2 inhibitors in post-AMI patients, including non-diabetic individuals; (2) evaluate the independent associations of SUA reduction and renal functional change with MI recurrence; and (3) employ causal mediation analysis to compare the relative contribution of two clinically measurable pathways with the observed post-AMI outcome pattern.

These two pathways were selected because they are both biologically plausible, mechanistically interconnected, and simultaneously influenced by SGLT2 inhibition, making them particularly suitable for comparative evaluation within a mediation framework.

Given that randomized data on recurrent MI-specific benefit after AMI remain heterogeneous, the present study was not designed to establish a definitive clinical efficacy signal, but rather to explore whether two biologically plausible pathways (urate reduction and renal functional preservation) differ in their relative contributions to post-AMI recurrent ischemic risk within an observational, hypothesis-generating framework. We hypothesized that, within a post-AMI observational cohort, renal functional changes would demonstrate a stronger association with recurrent MI risk than SUA reduction when both pathways were evaluated simultaneously within a mediation framework.

## 2. Materials and Methods

### 2.1. Study Design and Population

This study was designed as a retrospective observational cohort study based on routinely collected clinical data from consecutive patients hospitalized for AMI who underwent percutaneous coronary intervention at the Institute of Cardiovascular Diseases, Timișoara, Romania, a tertiary cardiovascular referral center. Patients were enrolled during the index hospitalization and followed through routine clinical assessments and rehospitalization records between January 2023 and October 2025. Data were collected from the electronic registry of the hospital.

Inclusion criteria were as follows: (1) hospitalization for AMI, (2) availability of baseline and follow-up SUA and creatinine measurements, and (3) documented follow-up for recurrent events.

**Figure 1:**
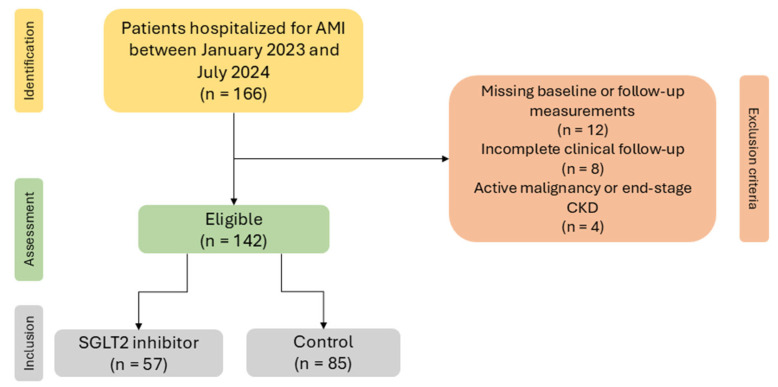
STROBE flow diagram of participants.

Exclusion criteria comprised the following: (a) missing baseline or follow-up measurements of SUA, creatinine, or other key laboratory parameters; (b) incomplete clinical follow-up precluding ascertainment of recurrent events; and (c) significant concomitant conditions precluding reliable interpretation of biomarker changes, such as active malignancy or end-stage chronic kidney disease (CKD) requiring dialysis. A patient flow diagram conforming to STROBE reporting guidelines [15] is provided in Figure 1, specifying the number of patients screened, excluded with documented reasons, and included in the final analysis.

A total of 142 patients met inclusion criteria and were included in the final analysis. SGLT2 inhibitor therapy was initiated during hospitalization or at discharge according to clinician judgment and standard practice. Molecules utilized were dapagliflozin 10 mg/day or empagliflozin 10 mg/day, but, given the limited subgroup sizes, exposure was analyzed at the class level. Patients were categorized into two groups: SGLT2 inhibitor users (*n* = 57) and controls (*n* = 85). Both diabetic and non-diabetic patients were included to enable evaluation of SGLT2 inhibitor effects independent of glycemic status.

This study was conducted in accordance with the Declaration of Helsinki and institutional standards for retrospective clinical research using anonymized data (Ethics Committee approval nr. 14/10.01.2024, 10 January 2024). Given the retrospective design and exclusive use of de-identified data, individual informed consent was waived, per institutional review board policy.

### 2.2. Clinical and Laboratory Assessment

Demographic, clinical, and laboratory data were collected from electronic medical records. Baseline variables included age, sex, presence of T2DM, renal function, and concomitant medication use (including loop/thiazide diuretics and allopurinol therapy). Laboratory parameters were recorded at baseline (index hospitalization) and at follow-up, including serum uric acid (SUA), hemoglobin, triglyceride-glucose index (TyG) and neutrophil-to-lymphocyte ratio (NLR).

Renal function was assessed using serum creatinine, and baseline estimated glomerular filtration rate (eGFR) was calculated using the CKD-EPI 2021 equation [16]. Albuminuria was not available in the retrospective dataset and, therefore, could not be incorporated into the analysis. Changes in biomarkers between baseline and follow-up were calculated as absolute differences (Δ), defined as follow-up value—baseline value. A reduction in uric acid was expressed as ΔUA, with negative ΔUA values indicating a decrease.

### 2.3. Study Endpoints

The primary endpoint was the change in SUA from baseline to follow-up (ΔUA). The secondary endpoint was recurrence of MI during follow-up, defined as rehospitalization with a primary diagnosis of acute MI according to the Fourth Universal Definition of Myocardial Infarction criteria [17]. Recurrent ACS events, including unstable angina requiring hospitalization, were also recorded. Subgroup analyses were performed, stratified by diabetes status and ACS recurrence.

### 2.4. Statistical Analysis

All statistical analyses were performed using R statistical software version 4.5.1 (R Foundation for Statistical Computing, Vienna, Austria). A two-sided *p*-value < 0.05 was considered statistically significant for all analyses.

Distribution normality was assessed using the Shapiro–Wilk test. Continuous variables with normal distribution are presented as mean ± standard deviation (SD); variables with non-normal distribution are reported as median [interquartile range, Q1–Q3]. For variables where both parametric and non-parametric descriptors are informative (e.g., age, eGFR), both formats are reported with a corresponding footnote to ensure transparency. Categorical variables are expressed as counts and percentages. Between-group comparisons were performed using the Student’s *t*-test or Wilcoxon rank-sum test for continuous variables and χ^2^ or Fisher’s exact test for categorical variables, as appropriate.

Linear regression analysis was used to evaluate predictors of ΔUA and change in creatinine. Multivariable linear regression models included baseline uric acid, age, sex, diabetes status, eGFR, loop/thiazide diuretic use, and allopurinol therapy.

To address the physiological correlation between uric acid and creatinine, both being dependent on renal function, the variance inflation factor (VIF) was calculated for all predictor variables in multivariable models. All VIF values were below the conventional threshold of 5.0, confirming the absence of problematic multicollinearity and supporting the simultaneous inclusion of both biomarkers in regression models.

Multivariable logistic regression analysis was performed to identify independent predictors of MI recurrence. Variables included in the models were SGLT2 inhibitor use, ΔUA, change in creatinine (ΔCr), ΔNLR, change in hemoglobin (ΔHb), age, sex, diabetes status, and eGFR. Results are reported as regression coefficients (β), odds ratios (OR), and their 95% confidence intervals (CI). Interaction analyses were conducted to evaluate potential effect modification by diabetes status and recurrent MI. Interaction terms were included in regression models and assessed using likelihood ratio tests.

Given the physiological interdependence between uric acid and renal function, interaction and collinearity were assessed, and both variables were included in multivariable models. However, formal joint-mediator models were limited by sample size.

To explore potential mechanistic pathways linking SGLT2 inhibitor therapy with MI recurrence, causal mediation analyses were performed using the mediation package in R [19]. The following two mediation models were evaluated:

Model 1: The renal pathway: SGLT2 inhibitor therapy → change in creatinine (ΔCr) → recurrent MI.
Model 2: The uric acid pathway: SGLT2 inhibitor therapy → SUA reduction (ΔUA) → recurrent MI.

Mediator models were fitted using linear regression, and outcome models used logistic regression adjusted for age, sex, diabetes status, and eGFR. Mediation effects were estimated using nonparametric bootstrap methods with 1000 simulations. The average causal mediation effect (ACME), average direct effect (ADE), total effect, and proportion mediated were calculated for each pathway. Causal mediation analysis relies on the assumption of no unmeasured confounding among exposure, mediator, and outcome (sequential ignorability). Given the observational design, this assumption cannot be fully verified.

Propensity score matching was not performed because of the modest sample size and the risk of further reducing statistical power and event counts. Instead, confounding was addressed through multivariable adjustment including clinically relevant baseline covariates (age, sex, diabetes status, eGFR, baseline uric acid, concomitant medication, and biomarker changes, as appropriate to each model).

As a sensitivity analysis, an extended multivariable model was constructed to account for surrogate markers of alternative cardioprotective mechanisms. These included hemoconcentration (Δhemoglobin), inflammatory status (ΔNLR), metabolic profile (diabetes and TyG index), and RAAS-related therapies (ACEi/ARB/ARNI and mineralocorticoid receptor antagonists).

### 2.5. Post Hoc Power Analysis

Given the relatively modest sample size (*n* = 142), a post hoc power analysis was conducted to evaluate whether the study possessed sufficient statistical power to detect the total effect of SGLT2 inhibitor therapy on MI recurrence. Using the observed MI recurrence rates (16% in the SGLT2 inhibitor group vs. 13% in controls), a significance level of α = 0.05, and the respective sample sizes (SGLT2 inhibitor = 57, control = 85), the estimated statistical power for detecting the total effect was approximately 18–22%. This confirms that the study was underpowered for the secondary endpoint of MI recurrence as a direct (total) effect. Importantly, the indirect (mediated) effects were detectable given their larger effect sizes relative to the sample’s variance structure. This limitation is explicitly addressed in Section 4.4 (Limitations) and supports the characterization of this analysis as hypothesis-generating, rather than confirmatory.

Because of the retrospective design, no formal a priori sample size calculation was performed. The study included all consecutive eligible patients meeting inclusion criteria during the study period. To better contextualize statistical precision, we performed a post hoc power analysis for the recurrent MI endpoint.

## 3. Results

### 3.1. Study Population and Baseline Characteristics

A total of 142 patients were included in the final analysis. Among them, 57 (40.1%) received an SGLT2 inhibitor and 85 (59.9%) served as controls. T2DM was present in 67 patients (47.2%), while 75 (52.8%) were non-diabetic. During follow-up, 51 patients (35.9%) experienced at least one rehospitalization for recurrent ACS, of whom 20 (14.1%) had confirmed recurrent MI. Baseline demographic, clinical, and laboratory characteristics were comparable between groups and are summarized in Table 2.

### 3.2. Effect of SGLT2 Inhibitors on Serum Uric Acid

**Figure 2:**
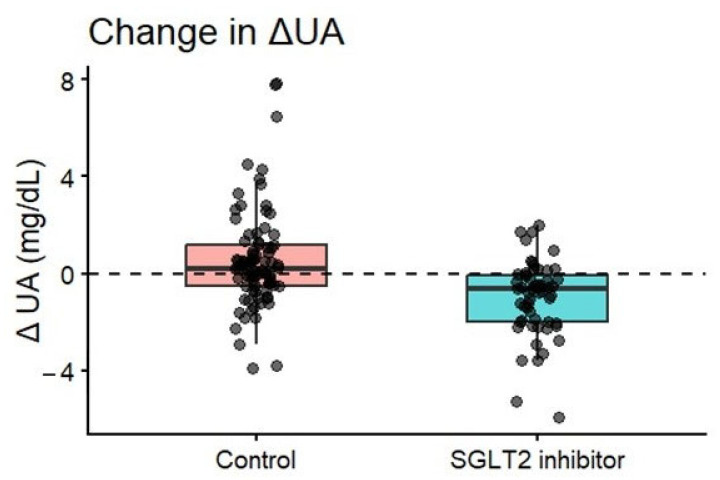
Distribution of change in serum uric acid levels (ΔUA), stratified by treatment group. Box plots represent median, interquartile range, and range. Controls exhibited a significant increase (ΔUA = +0.56 mg/dL), whereas SGLT2 inhibitor users showed a significant decrease (ΔUA = −0.99 mg/dL). Between-group comparison *p* < 0.001 (Wilcoxon rank-sum test).

Baseline SUA levels were comparable between groups (control: 5.60 ± 1.70 mg/dL vs. SGLT2 inhibitor: 5.94 ± 1.91 mg/dL; *p* = 0.28). During follow-up, the control group demonstrated a significant increase in SUA (ΔUA = +0.56 mg/dL, *p* = 0.014), whereas patients treated with SGLT2 inhibitors showed a marked reduction (ΔUA = −0.99 mg/dL, *p* < 0.001). The between-group difference in ΔUA was highly significant (mean difference: 1.55 mg/dL, 95% CI 0.95–2.14; *p* < 0.001) (Figure 2).

**Figure 3:**
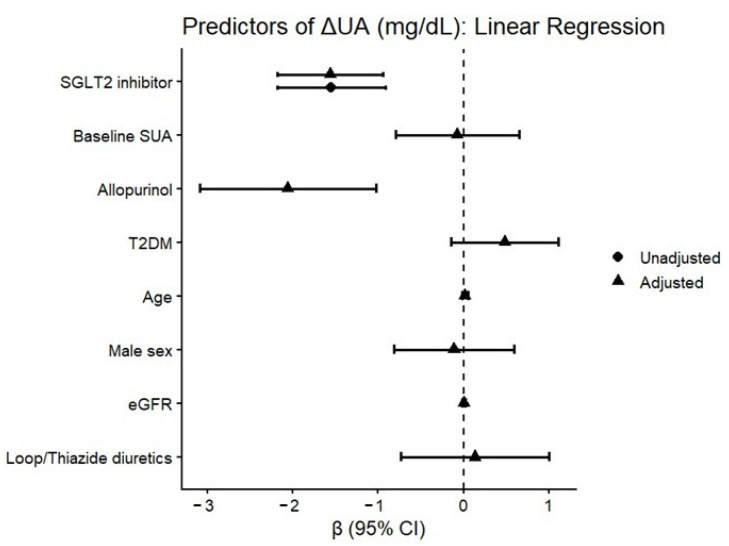
Forest plot of multivariable linear regression coefficients for predictors of ΔUA. SGLT2 inhibitor therapy was independently associated with SUA reduction after adjustment for baseline SUA, age, sex, T2DM, eGFR, diuretics, and allopurinol. Horizontal lines represent 95% CI.

In unadjusted linear regression analysis, SGLT2 inhibitor therapy was associated with a significant reduction in SUA (β = −1.55 mg/dL, 95% CI −2.18 to −0.92; *p* < 0.001). This association remained significant after multivariable adjustment for baseline SUA, age, sex, T2DM, eGFR, loop/thiazide diuretics, and allopurinol therapy (adjusted β = −1.43 mg/dL, 95% CI −2.04 to −0.83; *p* < 0.001). Higher baseline SUA and allopurinol therapy were also independently associated with greater SUA reduction (Figure 3).

### 3.3. Stratified Analysis by Diabetes Status

**Figure 4:**
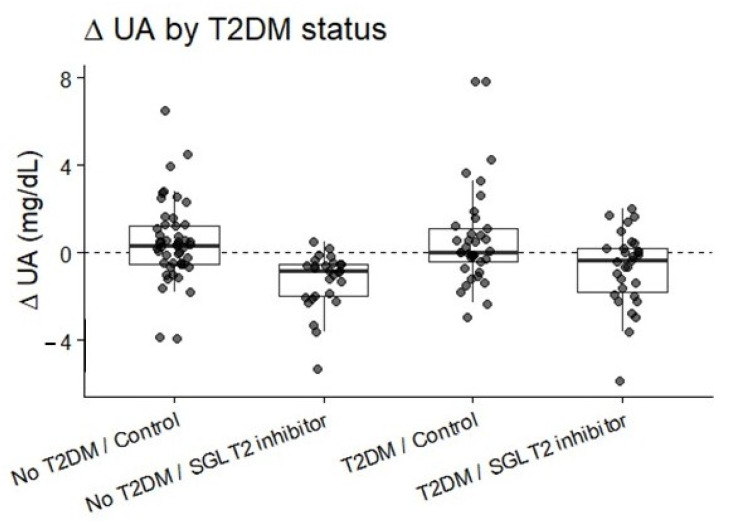
Distribution of ΔUA stratified by diabetes status and treatment group. SGLT2 inhibitor-associated SUA reduction was significant in both diabetic and non-diabetic subgroups.

**Figure 5:**
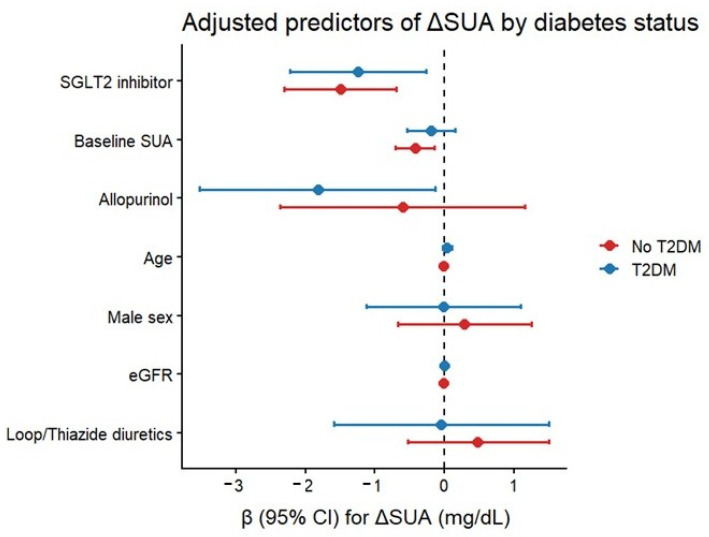
Forest plot of multivariable regression predictors of ΔUA, stratified by diabetes status. SGLT2 inhibitor therapy remained an independent predictor of SUA reduction in both subgroups.

When stratified by diabetes status, SGLT2 inhibitor therapy was associated with significant urate reduction in both subgroups. Among diabetic patients, SUA decreased significantly in the SGLT2 inhibitor group, whereas no significant change was observed in controls. Among non-diabetic patients, SGLT2 inhibitor users demonstrated a pronounced reduction in SUA, while controls exhibited only a mild, non-significant increase. Between-group differences in ΔUA remained significant in both diabetic and non-diabetic subgroups after multivariable adjustment (Figure 4 and Figure 5).

### 3.4. Interaction with Recurrent ACS

**Figure 6:**
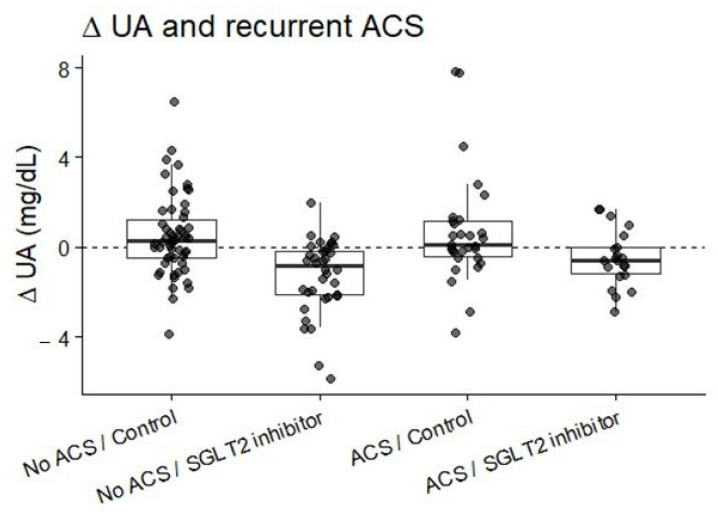
Distribution of ΔUA at follow-up, stratified by recurrence of ACS. SGLT2 inhibitor users showed consistent SUA reduction regardless of ACS recurrence status.

**Figure 7:**
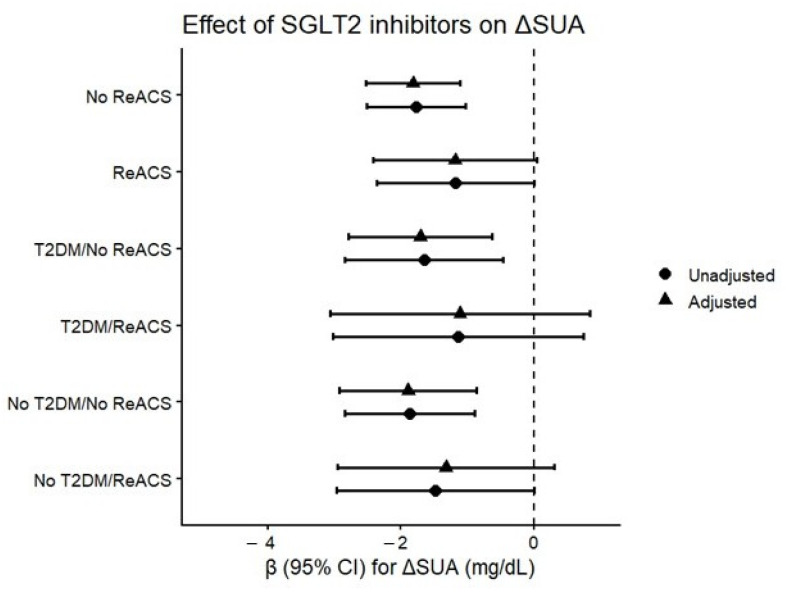
Forest plot of interaction analysis between SGLT2 inhibitor therapy and ACS recurrence on ΔUA. No significant interaction was observed (*p* = 0.37).

In an interaction model evaluating the influence of recurrent ACS on uric acid dynamics, SGLT2 inhibitor therapy remained significantly associated with SUA reduction in the overall cohort (β = −1.77 mg/dL, *p* < 0.001). No significant interaction was observed between SGLT2 inhibitor use and recurrent ACS status (*p* for interaction = 0.37), indicating that the urate-lowering effect of SGLT2 inhibitors was consistent regardless of rehospitalization status (Figure 6 and Figure 7).

Across all subgroup analyses, SGLT2 inhibitor therapy was consistently associated with SUA reduction, whereas SUA levels tended to increase in the control group.

### 3.5. Association Between Uric Acid Dynamics, Renal Function, and Myocardial Infarction Recurrence

In multivariable logistic regression analysis adjusted for age, sex, diabetes status, and eGFR, but without inclusion of renal functional change, greater reduction in SUA was associated with lower odds of MI recurrence (β = −0.25 per 1 mg/dL decrease; *p* = 0.041), corresponding to an approximate 22% relative risk reduction per unit decrease in SUA. No significant interaction was observed between SGLT2 inhibitor therapy and SUA reduction (*p* for interaction = 0.51), suggesting that the association between uric acid and MI recurrence was independent of treatment group.

However, when renal functional change (ΔCr) was incorporated into the multivariable model, the clinical picture changed substantially. Change in serum creatinine emerged as the strongest independent predictor of MI recurrence (β = 2.22, OR = 9.21 [95% CI 1.54–55.1]; *p* = 0.015), while the previously observed association between ΔUA and MI recurrence was attenuated and no longer reached statistical significance (β = −0.14, OR = 0.87 [95% CI 0.62–1.22]; *p* = 0.42). Older age also remained a significant predictor (*p* = 0.005). SGLT2 inhibitor therapy, changes in NLR, and hemoglobin were not independently associated with MI recurrence in this fully adjusted model (Table 3).

This pattern is more consistent with SUA acting as a surrogate marker of renal–metabolic status than with an independent contribution of SUA to recurrent MI in this cohort. The physiological coupling between uric acid renal clearance and glomerular function likely explains the initial apparent association, which was confounded by shared renal determinants.

A fully adjusted model, including surrogate markers of alternative cardioprotective pathways, was performed as a sensitivity analysis (Supplementary Table S1). The results were consistent with the primary analysis, with change in creatinine (ΔCr) remaining the only independent predictor of recurrent MI, while all other variables were not significantly associated with the outcome.

### 3.6. Causal Mediation Analysis

Causal mediation analysis was performed to formally test two competing mechanistic pathways linking SGLT2 inhibitor therapy with MI recurrence, decomposing the total treatment effect into indirect (mediated) and direct components.

Renal pathway (Model 1): A significant indirect effect of SGLT2 inhibitor therapy on MI risk was observed through changes in renal function (ΔCr). The average causal mediation effect (ACME) was statistically significant (*p* = 0.038), indicating that renal functional changes significantly mediated the association between SGLT2 inhibitor use and MI recurrence.

Uric acid pathway (Model 2): Initial mediation analysis without renal adjustment suggested a potential indirect effect through uric acid reduction (ACME = −0.057, 95% CI −0.121 to −0.0006; *p* = 0.044). However, when concurrently adjusted for renal function change, uric acid reduction was no longer a significant mediator (ACME *p* = 0.462). This finding suggests that the apparent uric acid-related pathway may largely reflect concurrent renal functional changes, rather than a clearly independent pathway.

The average direct effect (ADE) of SGLT2 inhibitors on MI risk was not statistically significant (*p* = 0.19), consistent with the limited statistical power for detecting total effects in this sample size, as documented in the post hoc power analysis (Section 2.5).

### 3.7. Integrated Mechanistic Findings

Taken together, these results are more consistent with renal functional change than with independent uric acid reduction as the main pathway associated with recurrent MI in this cohort. Although SGLT2 inhibitors significantly reduced SUA levels, an effect that was robust, consistent across diabetic and non-diabetic subgroups, and independent of ACS recurrence, uric acid reduction did not independently mediate MI risk after accounting for renal functional changes. These findings are consistent with a predominantly renal–metabolic framework for interpreting the observed associations in patients treated with SGLT2 inhibitors following AMI.

Baseline TyG values were comparable between groups. During follow-up, TyG decreased in both groups, with a numerically greater reduction observed in the SGLT2 inhibitor group (ΔTyG −0.73 ± 1.24 [−0.52; −0.98 to −0.06] vs. −0.41 ± 0.80 [−0.35; −0.83 to 0.09]); however, the between-group difference did not reach statistical significance (*p* = 0.16). In univariate logistic regression, ΔTyG was not associated with recurrent MI (β = 0.26, *p* = 0.406). Moreover, inclusion of ΔTyG in the fully adjusted multivariable model did not materially alter the independent association between renal functional change and MI recurrence.

Importantly, incorporation of the TyG index as a surrogate marker of insulin resistance did not modify the overall mechanistic pattern observed in this study. Although a numerical improvement in TyG was observed among SGLT2 inhibitor-treated patients, ΔTyG was not independently associated with recurrent MI and did not attenuate the renal-mediated pathway in multivariable analyses. These findings suggest that insulin resistance does not represent the dominant mechanism linking SGLT2 inhibition with cardiovascular outcomes in this post-AMI population.

The persistence of the renal-mediated pathway, despite adjustment for both uric acid dynamics and insulin resistance, supports a predominantly renal–metabolic pattern of association. Within this framework, uric acid reduction appears to reflect improved renal handling and overall metabolic status, rather than functioning as an independent causal determinant of recurrent ischemic events. Overall, our results indicate that the observed associations were more consistent with a renal-mediated pathway within the variables captured in this dataset and the biologically coherent pathway associated with cardiovascular protection following AMI in patients treated with SGLT2 inhibitors.

## 4. Discussion

The present study provides a structured comparative analysis of two biologically plausible mechanistic pathways through which SGLT2 inhibitors may confer cardioprotective effects following ACS. Using causal mediation analysis, a methodology that extends beyond conventional regression in its capacity to formally decompose treatment effects into direct and indirect components, we found that the observed association pattern was more consistent with renal functional preservation than with uric acid reduction as the principal pathway captured by our models, rather than through uric acid per se.

### 4.1. SGLT2 Inhibitors and Uric Acid: A Robust but Mechanistically Complex Effect

A positive association was observed among patients with AMI. Several studies have demonstrated that high uric acid levels are associated with more severe coronary artery involvement, larger infarct size, greater risk of acute plaque rupture, and higher in-hospital and long-term mortality in acute settings [34,37,38,39,40,41,43,45,48]. This suggests that SUA may play a more prominent role in early atherosclerotic stages, whereas, in more advanced disease, other factors may outweigh its impact [37]. Being linked to the atherogenic process, uric acid promotes the generation of reactive oxygen species (ROS), leading to endothelial dysfunction, low-density lipoprotein (LDL) oxidation, and activation of inflammatory pathways. Elevated intracellular uric acid further enhances the expression of vasoconstrictive mediators (angiotensin II, thromboxane, and endothelin-1) while amplifying inflammation through upregulation of C-reactive protein and NF-κB. In addition, uric acid promotes platelet activation, adhesion, and aggregation, and reduces nitric oxide bioavailability, contributing to endothelial impairment and the progression of atherosclerosis [38].

SUA levels may also be influenced by commonly used cardiovascular medication. Low-dose aspirin decreases urate excretion by approximately 15%, whereas high-dose aspirin may exert a uricosuric effect. Loop diuretics and thiazide agents are also frequent causes of hyperuricemia, mainly through volume depletion and impaired tubular urate secretion due to competition for renal organic anion transporters, with reported rates ranging from 6% to 21% [38].

Insulin resistance and hyperuricemia are tightly interconnected metabolic processes, and SGLT2 inhibitors have consistently demonstrated improvements in insulin sensitivity and reductions in hyperinsulinemia. Accordingly, it is biologically plausible that part of the observed uric acid reduction may reflect upstream improvements in insulin resistance, rather than a purely uricosuric effect [49,50,51].

To address this potential pathway, we incorporated the TyG index as a validated surrogate marker of insulin resistance [49]. Although TyG showed a numerical reduction in the SGLT2 inhibitor group, it was not independently associated with recurrent MI and did not materially alter the renal-mediated association observed in our multivariable and mediation analyses. These findings suggest that insulin resistance, at least as captured by TyG in this cohort, is unlikely to represent the primary mechanistic pathway linking SGLT2 inhibition with post-AMI cardiovascular outcomes.

Notably, when renal functional change was included in the mediation framework, the apparent uric acid-mediated pathway was attenuated, whereas the renal-mediated pathway remained statistically significant. This pattern supports the concept that renal hemodynamic and tubular mechanisms may constitute a more proximal determinant of cardiovascular risk reduction after AMI.

Although direct measures of insulin sensitivity (e.g., HOMA-IR or clamp-derived indices) were not available, the persistence of the renal-mediated association across both diabetic and non-diabetic subgroups further supports a mechanism that extends beyond purely glycemic or insulin-driven effects. Future prospective studies incorporating direct assessments of insulin sensitivity are warranted to further disentangle these interrelated metabolic and renal pathways.

Sodium–glucose cotransporter-2 (SGLT2) inhibitors have emerged as a key therapeutic class with proven benefits in HF and CKD, extending beyond glycemic control [52,53]. A consistent metabolic effect observed across clinical trials is a reduction in SUA, presumably mediated by an indirect uricosuric mechanism. By inducing glycosuria, SGLT2 inhibitors reduce proximal tubular urate reabsorption, partly through transporter modulation (e.g., GLUT9) and glycosuria-related changes in renal urate handling. This effect is directly related to the degree of glycosuria, resulting in a 3–5% increase in urinary urate excretion and a typical SUA reduction of approximately 0.8–1.6 mg/dL [38].

Our findings support the presence of a clinically meaningful and statistically robust association between SGLT2 inhibitor therapy and lower SUA levels (ΔUA = −0.99 mg/dL), an effect that persisted after multivariable adjustment and was consistent across diabetic and non-diabetic subgroups. These results are concordant with prior evidence from post hoc analyses of EMPA-REG OUTCOME and CREDENCE, which reported SUA reductions of 0.8–1.2 mg/dL with empagliflozin and canagliflozin respectively [54,55]. The demonstration of this uricosuric effect in non-diabetic post-ACS patients extends the existing evidence base and supports the hypothesis that SGLT2 inhibitor-mediated urate lowering operates through direct renal tubular mechanisms (URAT1/GLUT9 competition with glycosuria), rather than through glycemia-dependent pathways [36].

Importantly, the urate-lowering effect was independent of ACS recurrence status (*p* for interaction = 0.37), reinforcing the consistency of this metabolic effect across diverse clinical subgroups within the post-ACS population.

### 4.2. The Surrogate Marker Hypothesis: Resolving the Apparent Mediation Paradox

A central finding of this study is the attenuation of the SUA–MI association upon incorporation of renal functional change into multivariable models. In the initial model excluding ΔCr, each 1 mg/dL decrease in SUA was associated with an approximately 22% reduction in MI risk (β = −0.25; *p* = 0.041). However, when ΔCr was added as a covariate, the association between ΔUA and MI recurrence lost significance (β = −0.14; *p* = 0.42), while creatinine change became the dominant predictor (β = 2.22; *p* = 0.015).

This observation is physiologically coherent. SUA levels are fundamentally determined by the balance between hepatic purine metabolism and renal urate clearance, a process that is itself governed by glomerular filtration rate and tubular transport capacity. SGLT2 inhibitors modulate both sides of this equation: they enhance glycosuria-driven urate excretion through URAT1/GLUT9 competition, while simultaneously restoring tubuloglomerular feedback and reducing glomerular hyperfiltration [46,47]. Consequently, the observed SUA reduction in SGLT2 inhibitor-treated patients may reflect improvements in renal hemodynamics, rather than representing an independent cardioprotective mechanism [34].

The mediation analysis provides quantitative evidence that is consistent with this “surrogate marker” hypothesis: when the renal pathway was explicitly modeled, its mediation effect was significant (ACME *p* = 0.038), whereas the uric acid pathway’s significance was abolished after renal adjustment (ACME *p* = 0.462). This does not negate the clinical utility of SUA monitoring, as it may serve as a readily accessible biomarker for tracking the renal–metabolic response to SGLT2 inhibitor therapy, but it cautions against attributing independent causal weight to uric acid changes when interpreting cardiovascular outcomes [39].

This interpretation resonates with several converging lines of evidence. In the FREED trial, febuxostat-mediated xanthine oxidase inhibition achieved substantial SUA reductions, but did not translate into unambiguous cardiovascular benefit, suggesting that uric acid lowering per se may be insufficient for cardioprotection [56]. Moreover, Mendelian randomization studies using genetic instruments for SUA have increasingly questioned the causal role of uric acid in cardiovascular disease, with several analyses finding no significant effect after accounting for renal function and metabolic confounders [57]. Collectively, these data support the notion that the cardiovascular risk traditionally attributed to hyperuricemia may be substantially confounded by underlying renal dysfunction. Residual confounding remains possible, particularly in the mediator–outcome relationship, which represents a key limitation of mediation analysis in observational datasets.

### 4.3. Renal Function as the Strongest Association Among Measured Pathways

The emergence of ΔCr as the strongest independent predictor of MI recurrence (β = 2.22; OR = 9.21; *p* = 0.015) underscores the importance of renal functional preservation in post-ACS cardiovascular risk stratification. This finding is consistent with the well-established cardiorenal axis paradigm, whereby renal deterioration amplifies neurohormonal activation, sodium retention, volume overload, and systemic inflammation, all of which accelerate atherosclerotic progression and myocardial vulnerability [58,59].

The potential mechanisms through which SGLT2 inhibitors may preserve renal function, and thereby reduce cardiovascular risk, include the following: (a) restoration of tubuloglomerular feedback via natriuresis at the macula densa, leading to afferent arteriolar vasoconstriction and reduced intraglomerular pressure [47]; (b) hemodynamic benefits, including reduced preload and afterload through osmotic diuresis and natriuresis [60]; (c) anti-inflammatory effects, mediated through suppression of the NLRP3 inflammasome pathway [61]; and (d) metabolic reprogramming favoring ketone body utilization as a more efficient myocardial fuel substrate [62].

Clinical implication: These data suggest that monitoring renal function trajectory (creatinine, eGFR) should be prioritized in the longitudinal risk assessment of post-ACS patients receiving SGLT2 inhibitor therapy. A favorable renal functional trajectory may serve as a more reliable clinical indicator of cardiovascular benefit than SUA changes alone. Conversely, worsening renal function despite SGLT2 inhibitor therapy should prompt re-evaluation of overall cardiovascular risk and therapeutic adequacy.

Importantly, the inclusion of surrogate markers reflecting alternative cardioprotective mechanisms did not materially alter the observed associations. Even after adjustment for hemoconcentration, inflammatory markers, metabolic status, and RAAS-related therapies, the renal-associated signal remained the only independent predictor of recurrent myocardial infarction. These findings suggest that the observed pattern is robust to adjustment for competing mechanistic pathways within the limits of available clinical proxies.

### 4.4. Limitations

Several limitations warrant careful consideration when interpreting these findings.

First, the retrospective observational design precludes definitive causal inference, notwithstanding the use of causal mediation analysis. While this methodology provides stronger mechanistic evidence than conventional regression by formally testing mediation pathways, it remains dependent on the sequential ignorability assumption, specifically that there are no unmeasured confounders of the mediator–outcome relationship. The mediation results should therefore be interpreted as hypothesis-generating, rather than confirmatory of causal mechanisms. Second, the sample size (*n* = 142) confers limited statistical power for detecting the total effect of SGLT2 inhibitors on MI recurrence (estimated power: 18–22%; see Section 2.5). While the indirect (mediated) effects were detectable due to their larger effect sizes, a larger prospective cohort would be needed to validate these pathway-specific findings and to detect potentially smaller total effects with adequate power. Third, we did not have access to serial echocardiographic data (e.g., left ventricular ejection fraction trajectories) or contemporary cardiac biomarkers (e.g., NT-proBNP, high-sensitivity troponin), which would have enabled a more comprehensive mechanistic assessment of the cardiorenal interplay under SGLT2 inhibitor therapy. Fourth, the single-center design at a tertiary cardiovascular referral center may limit generalizability, although the use of consecutive patient enrollment and a broad ACS spectrum (both ST elevation and non-ST elevation MI) mitigate selection bias to some extent. Fifth, while the VIF analysis confirmed absence of problematic multicollinearity (all VIF < 5.0), the inherent physiological correlation between uric acid and creatinine represents an intrinsic challenge in fully separating these two pathways in any observational framework. Although uric acid and renal function are closely interrelated, the present analysis was not powered for formal joint-mediator modeling, which should be addressed in future studies. Future studies employing instrumental variable approaches, Mendelian randomization designs, or randomized controlled trials with mechanistic sub studies could help further disentangle these interdependent mechanisms. The cohort did not include a dedicated comparator population with hospitalization for HF; therefore, the present analysis cannot distinguish whether the observed biomarker–outcome relationships are AMI-specific or reflect broader cardiorenal effects already described in HF populations.

An important limitation of this study is that the observed associations are exploratory and based on indirect clinical surrogate markers, rather than direct mechanistic measurements. Although we attempted to account for several alternative pathways using available proxies, the present analysis does not provide a formal methodological framework to exclude other potential pleiotropic effects of SGLT2 inhibitors. Therefore, the findings should not be interpreted as evidence that the observed associations are exclusively mediated through the investigated pathways, nor do they rule out the contributions of other established or unknown mechanisms. The present study cannot exclude the involvement of alternative or parallel pathways, and the results should not be interpreted as isolating or confirming a single dominant causal mechanism. Rather, these findings should be considered hypothesis-generating and reflective of association patterns within the constraints of the available data.

### 4.5. Future Directions

Prospective multicenter studies with larger sample sizes, serial biomarker assessment, and randomized allocation of SGLT2 inhibitors vs. active comparators are warranted to confirm the primacy of the renal pathway identified in the present analysis. Integration of novel kidney injury biomarkers (e.g., KIM-1, NGAL, uromodulin), alongside established cardiac biomarkers (NT-proBNP, high-sensitivity troponin) and mechanistic imaging modalities (cardiac MRI, renal Doppler ultrasonography), could further refine the understanding of cardiorenal interplay under SGLT2 inhibitor therapy [63,64].

Additionally, Mendelian randomization approaches using genetic instruments for SUA levels and renal function could complement the observational mediation framework applied in this study, providing an independent line of evidence regarding the causal architecture of these interrelated pathways. Subgroup analyses by specific SGLT2 inhibitor agent (empagliflozin, dapagliflozin, canagliflozin), and by baseline renal function strata, could also elucidate potential differential effects across patient populations.

Our findings should, therefore, be interpreted as mechanistic associations within a retrospective observational cohort, not as evidence that SGLT2 inhibitors definitively prevent recurrent MI through a confirmed causal pathway.

## 5. Conclusions

This retrospective cohort study found that SGLT2 inhibitor therapy was associated with significant, consistent, and diabetes-independent reduction in SUA following AMI. However, causal mediation analysis suggests that the observed association pattern is more likely mediated through renal functional preservation, rather than through uric acid reduction per se.

SUA dynamics function primarily as a surrogate marker of underlying renal–metabolic processes, rather than as an independent causal pathway to cardiovascular protection. These findings support the clinical prioritization of renal function monitoring (creatinine, eGFR) in the management of post-AMI patients receiving SGLT2 inhibitor therapy and underscore the importance of the cardiorenal axis within the observed association in mediating the therapeutic benefits of this drug class.

Given the retrospective observational design and the modest sample size, these results should be interpreted as hypothesis-generating. The mediation findings warrant prospective validation in larger, multicenter cohorts with adequate statistical power and serial biomarker assessment to definitively establish the primacy of the renal–metabolic pathway in SGLT2 inhibitor-mediated cardiovascular protection after AMI. These findings should not be interpreted as excluding other pleiotropic mechanisms of SGLT2 inhibitors, but rather as identifying the renal pathway as the most consistent association among the variables captured in this dataset.

## Figures and Tables

**Table 1:** UA values associated with ischemic events.

Author and Year	Ischemic Outcomes	SUA Cut-Off
Akpek et al. (2011) [40]	Poor coronary flow In-hospital MACE	5.4 mg/dL
Akgul et al. (2014) [41]	6-month all-cause mortality	5.7 mg/dL
Braga et al. (2016) [42]	Coronary events	7 mg/dL
Perticone et al. (2023) [43]	Coronary events	5.3 mg/dL (male) 5.2 mg/dL (female)
Talpur et al. (2023) [44]	Non-fatal MI	7 mg/dL
Virdis et al. (2020) [45] The URRAH project	Fatal MI	5.7 mg/dL

**Table 2:** Baseline characteristics of the study population, stratified by SGLT2 inhibitor exposure.

Variable	Control (*n* = 85)	SGLT2 Inhibitor (*n* = 57)	*p*-Value ††
Age (years) †	62 ± 11 [62; 52–71]	61 ± 10 [60; 55–66]	0.69
Male sex, *n* (%)	61 (72%)	45 (79%)	0.43
T2DM, *n* (%)	37 (44%)	30 (53%)	0.3
Arterial hypertension, *n* (%)	72 (%)	47 (82.4%)	0.81
Obesity	30 (35.3%)	13 (22.8%)	0.13
Smoking	48 (56.5%)	34 (59.6%)	0.86
eGFR (mL/min/1.73 m^2^) †	75 ± 21 [74; 60–93]	79 ± 20 [79; 67–92]	0.22
Recurrent ACS, *n* (%)	30 (35%)	21 (37%)	0.86
Time to event (months)	3 [1–7]	5 [3–12]	0.17
Re-MI, *n* (%)	11 (13%)	9 (16%)	0.63
Time to event (months)	8 [5–14]	13 [10–19]	0.13
SUA baseline (mg/dL)	5.60 ± 1.70	5.94 ± 1.91	0.28
SUA follow-up (mg/dL) †	6.16 ± 2.35 [5.90; 4.50–7.20]	4.95 ± 1.71 [4.70; 4.00–5.50]	<0.001
ΔUA (mg/dL) †	+0.56 ± 2.04 [0.20; −0.50 to 1.20]	−0.99 ± 1.55 [−0.60; −2.00 to −0.10]	<0.001
ΔTyG index baseline †	9.06 ± 0.67	9.2 ± 0.71	0.21
	[8.89; 8.61–9.68]	[9.15; 8.69–9.74]	
TyG index follow-up †	8.65 ± 0.69	8.46 ± 1.14	0.73
	[8599; 8.16–9.09]	[8.60; 8.13–9.06]	
Loop/thiazide diuretics, *n* (%)	74 (87%)	47 (82%)	0.47
Allopurinol, *n* (%)	8 (9.4%)	6 (11%)	1
Time to SGLT2 inhibitor initiation (days)		4.2 ± 3.1 [3; 2–6]	
Dapagliflozin 10 mg/day	0%	40 (70.2%)	
Empagliflozin 10 mg/day	0%	17 (29.8%)	
STEMI	82 (96.5%)	52 (91.2%)	0.26
Multivessel CAD	26 (%)	59 (%)	0.85
Number of stents placed	2 [1–3]	2 [1–2]	0.8
CKD stage (KDIGO), *n* (%)			
G3a-G3b	22 (26%)	10 (17.5%)	0.3
ACEi/ARB/ARNI use, *n* (%)	60 (%)	45 (78.9%)	0.33
MRA use, *n* (%)	70 (81.2%)	50 (87.7%)	0.48
Beta-blocker use, *n* (%)	65 (%)	44 (77.2%)	1
Statin use, *n* (%)	85 (100%)	57 (100%)	1
LVEF at discharge (%)	40 [35–45]	39 [35–41]	0.19

† Variables with non-normal distribution (Shapiro–Wilk *p* < 0.05) are reported as mean ± SD [median; Q1–Q3]. Normally distributed continuous variables are reported as mean ± SD only. †† Wilcoxon rank-sum test for continuous variables; Fisher’s exact test for categorical variables. MRA = Mineralocorticoid receptor antagonist.

**Table 3:** Multivariable logistic regression model: independent predictors of recurrent myocardial infarction.

Variable	β	OR (95% CI)	*p*-Value	VIF
ΔCreatinine (mg/dL)	2.22	9.21 (1.54–55.1)	0.015	1.23
Age (years)	0.08	1.08 (1.02–1.15)	0.005	1.15
ΔUA (mg/dL)	−0.14	0.87 (0.62–1.22)	0.42	1.89
SGLT2 inhibitor use	−0.47	0.63 (0.19–2.06)	0.44	1.34
ΔNLR	0.03	1.03 (0.91–1.16)	0.68	1.08
ΔHemoglobin (g/dL)	−0.21	0.81 (0.54–1.22)	0.31	1.12
Diabetes (T2DM)	0.35	1.42 (0.44–4.58)	0.56	1.28
Sex (male)	−0.52	0.59 (0.17–2.12)	0.42	1.09
eGFR (mL/min/1.73 m^2^)	−0.01	0.99 (0.96–1.02)	0.55	1.45

VIF = variance inflation factor. All VIF < 5.0, confirming absence of problematic multicollinearity. OR = odds ratio; CI = confidence interval.

## Data Availability

The data presented in this study are available on request from the corresponding author. The data are not publicly available due to privacy restrictions governing patient health information under applicable Romanian and European data protection legislation (GDPR).

## Supplementary Materials

The following supporting information can be downloaded at: https://www.mdpi.com/article/10.3390/biomedicines14040842/s1, Table S1: Fully adjusted model including surrogate markers of alternative cardioprotective pathways.
