# Supplementary material for: Disentangling Uric Acid and Renal Pathways in SGLT2 Inhibitor Effects After Acute Myocardial Infarction: A Retrospective Mediation Analysis
Source: Biomedicines. 2026 Apr 7;14(4):842. doi: 10.3390/biomedicines14040842 (PMC13114264; doi:10.3390/biomedicines14040842)
Supplement: Supplementary file 1 [file biomedicines-14-00842-s001.zip › biomedicines-4199634-supplementary.pdf]

Supplementary Table S1. Fully adjusted model including surrogate markers of alternative cardioprotective pathways

**Variable**	**OR (95% CI)**	**p-value**
ΔCreatinine (mg/dL)	9.87 (1.63–69.52)	0.015
Age (years)	0.89 (0.82–0.96)	0.003
ΔUric Acid (mg/dL)	1.12 (0.82–1.55)	0.472
SGLT2 inhibitor use	2.50 (0.77–8.57)	0.133
ΔHemoglobin (g/dL)	1.18 (0.74–1.92)	0.485
ΔNLR	1.10 (0.97–1.28)	0.168
Diabetes (T2DM)	2.64 (0.73–10.34)	0.146
Sex (male)	0.86 (0.26–2.99)	0.798
eGFR (mL/min/1.73 m^2)	0.97 (0.93–1.00)	0.068
RAAS blockade	0.58 (0.18–1.97)	0.367
MRA use	0.73 (0.20–3.09)	0.643
TyG index (baseline)	0.59 (0.22–1.47)	0.268
